# Spatiotemporal evolution and driving factors of soil erosion function in a typical basin of the alpine region in the upper Yellow River

**DOI:** 10.3389/fpls.2025.1589848

**Published:** 2025-06-09

**Authors:** Xiaomei Kou, Qi Li, Zhen Song, Qin Yu, Baicheng Niu, Shan Wang, Kaifang Mu, Wenlin Du

**Affiliations:** ^1^ Power China Northwest Engineering Corporation Limited, Xi’an, China; ^2^ School of National Security and Emergency Management, Qinghai Normal University, Xining, China; ^3^ School of Geographical Science, Qinghai Normal University, Xining, China; ^4^ Key Laboratory of Mountain Hazards and Earth Surface Process, Institute of Mountain Hazards and Environment, Chinese Academy of Sciences, Chengdu, China; ^5^ Qinghai Yellow River Upstream Hydropower Development Co., Ltd., Longyangxia Power Generation Branch Hainan Tibetan Autonomous Prefecture, Xining, China

**Keywords:** InVEST model, soil erosion intensity, soil conservation function, Geodetector, upper Yollow River

## Abstract

In recent years, soil erosion has become increasingly severe in the Tibet Plateau, especially in the upper Yellow River Basin. Although numerous studies have been conducted on soil erosion in this region, most of them are limited to short time spans and fail to reflect the temporal variations of soil erosion at long-term scales. In this study, the spatiotemporal changes in soil erosion intensity and soil conservation function were evaluated using the InVEST model, and driving factors were identified using Geodetector in the Shagou River watershed from 1980 to 2020. The results showed that over the past 40 years, the Shagou River watershed mainly suffered mild erosion, the soil erosion intensity increased by 81.40% from 1980 to 2000, reaching a peak of approximately 1292.49 t/(km²·a). From 2000 to 2020, it decreased by 40.45% with 769.63 t/km²·a, indicating a clear improvement trend. From 1980 to 1990, the growth rate of soil conservation function was most significant, reaching 25.89%, while the growth rate from 2010 to 2020 was relatively small, only 6.23%. Over the past 40 years, the total soil conservation function increased by 1.12×10⁷ t, with a growth rate of 88.24%, reflecting the effectiveness of soil conservation measures. In addition altitudes, slopes, and land use types had significantly affected both on actual soil erosion intensity and soil conservation function. Geodetector analysis revealed that the primary factors influencing soil erosion intensity were vegetation cover and elevation, along with their two - factor interaction. Regarding the soil conservation function, the main influencing factors were elevation and precipitation, with the interaction dominated by elevation. This study should provide a theoretical basis and scientific reference for ecological protection and sustainable development in the alpine region of the upper Yellow River Basin.

## 1 Introduction

In recent years, climate warming and human activities have exacerbated soil erosion across the Tibetan Plateau in China, resulting in an increasingly sedimentation problem, particularly in the upper reaches of the Yellow River (Wang et al., 2024; Yu et al., 2024). This region, characterized by temperate semi-arid grasslands and arid desert grasslands (Mao et al., 2023), is subject to complex erosion processes driven by frequent fluctuations in wet and dry climatic conditions, resulting in severe soil erosion (Guo et al., 2024) and increased sediment transport to the Yellow River (Man et al., 2024). Annually, as much as 3×10^7^m^3^ of sediment from river tributaries, bank collapses, and quicksand was deposited in the Longyangxia Reservoir. These rivers are the primary sediment contributors to the upper reaches of the Yellow River (Zhao, 2019; Li et al., 2024). Given this context, research into soil erosion and its ecological implications on the Tibetan Plateau is critical for mitigating sediment loads and supporting the sustainable development of this region.

Soil is the foundation of ecosystem service and functionality (Li et al., 2022a). Its ecosystem conservation capacity plays a pivotal role in its service functions (Liu et al., 2019). Models are usually an important tool to assess soil conservation function. Among the models, the InVEST (Integrated Valuation of Ecosystem Services and Tradeoffs) model has superior accuracy and applicability in quantifying soil conservation function (Yang et al., 2022) that helps allow for intuitive and rational evaluations of soil conservation function (Guo et al., 2022). Additionally, the Geodetector provide a powerful tool that is widely used to investigate the attribution of spatial divergence and influence mechanisms (Wang and Xu, 2017; Gong et al., 2023; Liu et al., 2023; Chen et al., 2023).

In the middle and upper reaches of the Yellow River, many studies have been conducted to characterize soil erosion distribution, evaluate soil sensitivity (Wu and Wang, 2021; Xiao et al., 2021), and quantify ecological services (Yang et al., 2020, Yang et al., 2021a, Yang et al., 2021b, Yang et al., 2021c; Wei et al., 2022; Zhao et al., 2023; Xu et al., 2023; Liu et al., 2024; Qiao et al., 2024). However, most studies mainly focused on short-term time scales (Li et al., 2022b, Li et al., 2024). The long-term trends and driving mechanisms of soil erosion and soil conservation function, particularly in the upper alpine zones of the Yellow River Basin, remain insufficiently be explored. It is difficult to capture both the long - term cumulative impacts and spatial heterogeneity of soil erosion processes. Additionally, existing research often fails to effectively represent the nonlinear relationships between dynamic factors such as climate variability and vegetation dynamics and the intensity of erosion.

Therefore, this study focuses on the Shagou River watershed, a first-order tributary located in the alpine region of the upper of Yellow River. The research utilizes the InVEST model combined with the Geodetector to analyze the spatiotemporal characteristics of soil erosion and soil conservation function in the Shagou River watershed over the past 40 years. The study investigates the differentiation under different land use types, slope gradients, and elevation gradients. Specially, the objectives are to identify the spatiotemporal changes of soil erosion intensity and soil conservation function, explore the driving factors, and provide scientific recommendations for the high-quality development of the upper Yellow River basin, as well as the ecological protection and sustainable development of the Tibetan Plateau.

## 2 Materials and methods

### 2.1 Study area

**Figure 1:**
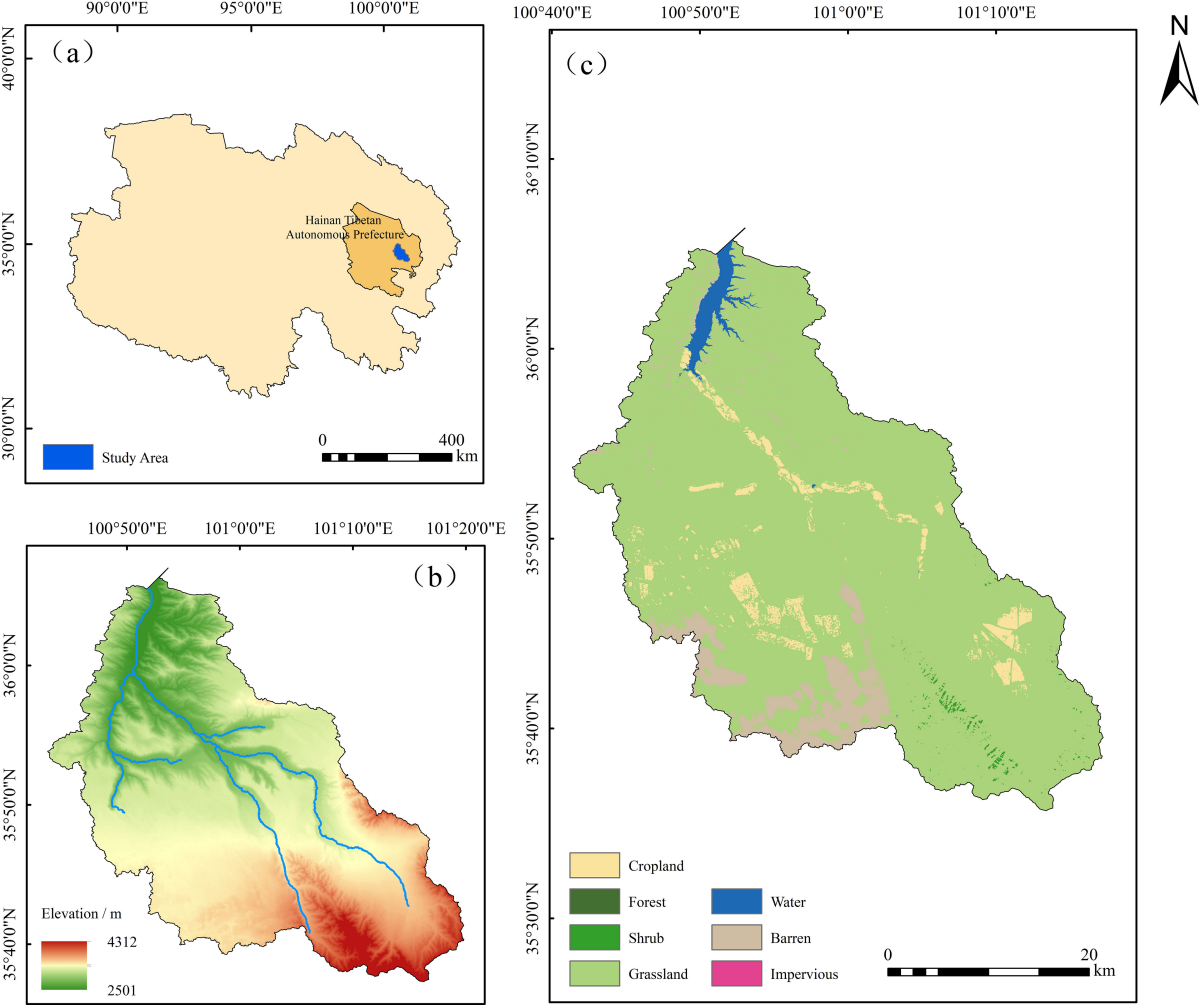
Location of the study area **(a)**, digital elevation model (DEM, **(b)**), and land use types of the Shagou River watershed **(c)**.

The Shagou River watershed is in Guinan County, Qinghai Province, China (**Figure 1**). The river runs from southeast to northwest, and goes to the Longyangxia Reservoir. The river is a first-order tributary of the Yellow River, with a watershed area of 1,531 km² and an elevation range of 2,501-4,312 m. This watershed exhibits a distinct topography, with higher elevations in the southeast and lower elevations in the northwest. Alpine meadows dominate the upper reaches, and transition to desertification in the central region. The primary soil type is sandy loam, while calcareous chestnut soils are prevalent in the Yellow River valley, particularly in its lower reaches.

This region features a typical plateau continental climate, characterized by a long Winter and short Summer. Precipitation and heat coincide seasonally, with a multi-year average annual rainfall of approximately 143 mm (Liu, 2017). The land use is primarily composed of grasslands, bare lands, and croplands.

### 2.2 Data sources

The datasets primarily include soil, land use types, DEM, meteorological, and vegetation cover. Specifically, the monthly precipitation data from 1980 to 2020 with a resolution of 1 km was obtained from the National Earth System Science Data Center (http://www.geodata.cn). Land use data from 1980 to 2020 was sourced from GlobelLand30 (http://globallandcover.com). The 30-m resolution DEM data was obtained from the Geospatial Data Cloud (https://www.gscloud.cn). Soil properties (i.e., HWSD soil properties data) and Normalized Difference Vegetation Index (NDVI) data were both acquired from the National Tibetan Plateau Science Data Center (https://data.tpdc.ac.cn).

### 2.3 InVEST model

The Sediment Delivery Ratio module within the InVEST model was utilized to establish a generalized methodology for quantifying soil erosion and sediment yield across diverse landscapes. The input data includes the soil erodibility factor (*K*), slope length factor (*L*) and slope gradient factor(*S*), rainfall erosive factor (*R*), vegetation cover and management factor (*C)*, and soil conservation measure factor (*P*).

#### 2.3.1 *K* factor

**Figure 2:**
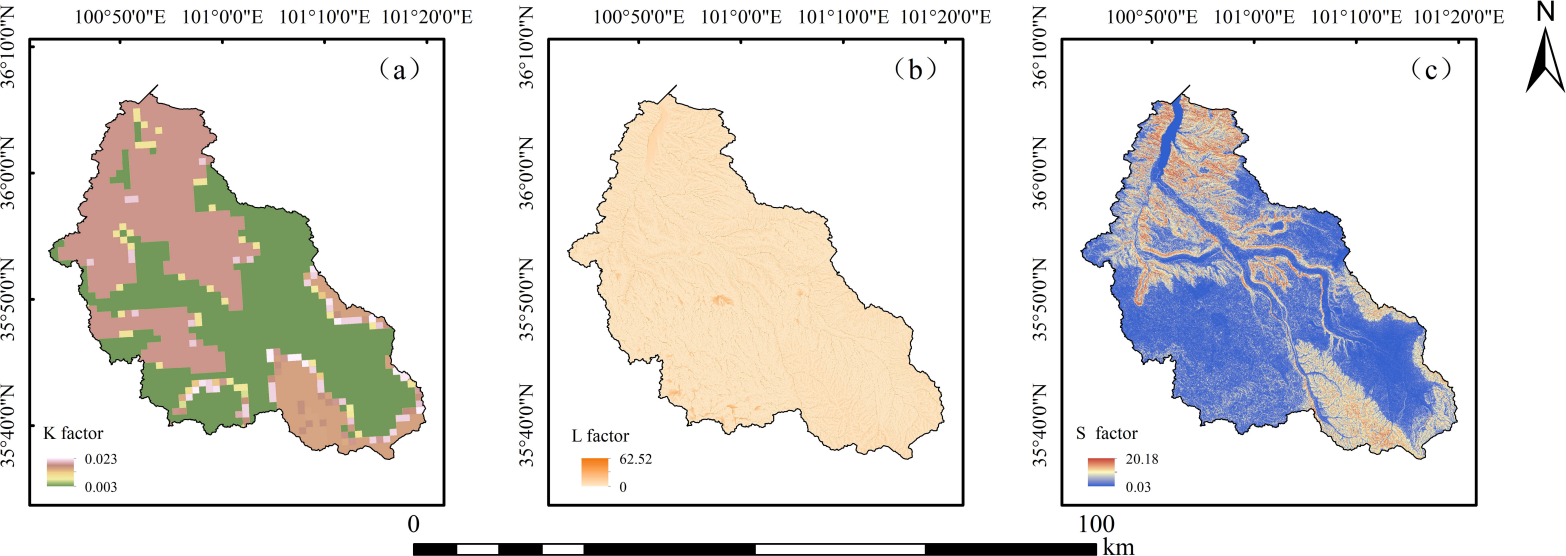
Soil erodibility *K* **(a)**, slope length *L* **(b)**, and slope degree *S* **(c)** of the Shagou River watershed.


The *K* factor was calculated using the Equation 1 proposed by Williams (1990) (**Figure 2a**):

(1) $$\begin{array}{c} K=\{0.2+0.3exp[-0.256SAN(\frac{1-SIL}{100})]\}{\left[\frac{SIL}{CLA+SIL}\right]}^{0.3}\\ \times \{1-\frac{0.25C}{C+exp(3.72-2.95C)}\}\\ \times [1-\frac{0.7(1-\frac{SAN}{100})}{\left(1-\frac{SAN}{100})+exp(-5.51+22.9(1-\frac{SAN}{100}\right)}] \end{array}$$

Where *K* is the soil erodibility factor [t·km^2^·h/(km^2^·MJ·mm)], *SAN*, *SIL*, and *CLA* denote the percentages of sand, silt, and clay in the soil, respectively; *C* is the soil organic carbon content. The calculation result *K* in US customary units should be multiplied by the conversion factor 0.1317 to obtain SI units.

#### 2.3.2 *L* and *S* factor

The DEM data was used to calculate the *L* and *S* factor. Considering that most areas in the Shagou River watershed feature slopes of less than 10°, the targeted topographic factor Equations 2, 3 proposed by Fu et al. (2015) was used to calculate *L* and *S* (**Figures 2b, c**):


(2) $$\begin{array}{c} L={\left(\frac{\lambda }{22.13}\right)}^{m};\\ m=(\begin{array}{cc} 0.2\; & \theta <0.5{}^{\circ}\\ 0.3\; & 0.5{}^{\circ}\leq \theta <1.5{}^{\circ}\\ 0.4\; & 0.5{}^{\circ}\leq \theta <3{}^{\circ}\\ 0.5\; & \theta >3{}^{\circ} \end{array}) \end{array}$$

(3) $$S=\{\begin{array}{cc} 10.8sin\;\theta +0.03 & \theta <5{}^{\circ}\\ 16.8sin\;\theta +0.5 & 5{}^{\circ}\leq \theta \leq 10{}^{\circ}\\ 21.9sin\;\theta +0.96 & \theta \geq 10{}^{\circ} \end{array}$$

Where *L* and *S* is the slope length and slope factor, respectively (dimensionless); *λ* denotes the horizontal projected slope length; *m* is the slope length index (also dimensionless); *θ* is the ground slope.

#### 2.3.3 *C* factor

The *C* factor was calculated using the following Equations 4, 5 suggested by Cai et al. (2000):


(4) $$f=\frac{NDVI-NDVI_{min}}{NDVI_{max}-NDVI_{min}}$$

(5) C={1,f=0 0.6508−0.3436lgf*100,0<f≤78.3%0,f>78.3% }

Where *f* is the vegetation cover (%), *NDVI* is the normalized vegetation index, whereas *NDVI_min_* and *NDVI_max_* refer to the minimum and minimum *NDVI* values, respectively.

#### 2.3.4 *R* factor

In this study, the Wischmeier’s monthly scale Equation 6 was utilized to obtain the *R* factor (Wischmeier and Mannering, 1969), as follows:


(6) $$R=\sum_{i=1}^{12}\left(1.734\times 10^{1.5\text{lɡ}\frac{P_{i}}{P}-0.8188}\right)$$

Where *P* is the annual precipitation (mm), *P_i_* refers to the precipitation in the month *i* (mm);*R* is the rainfall erosivity factor (MJ·mm)/(km^2^·h·a).

#### 2.3.5 *P* factor

This factor refers to the ratio of soil loss after implementing soil conservation measures to soil loss from planting downslope. In the study, the values of *P* in **Table 1** were sourced from the research of Li et al. (2022a).

**Table 1:** Values *P* factor for various land use types in the Shagou River watershed.

Land use	Cropland	Forest	Grassland	Water	Barren	Impervious	Shrub
*P* value	0.35	1	1	0	1	0	1

### 2.4 Actual soil erosion intensity

The Revised Universal Soil Loss Equation, which serves as the erosion module of the InVEST model, was employed to compute the actual soil erosion intensity. When soil and water conservation measures were not in place, the potential soil erosion was designated as *SL_0_*. In the context of this study, considering the implementation of soil conservation measures, the actual soil erosion intensity was denoted as *SL_t_*. (Equations 7–9) were utilized to determine the quantity of soil conservation:


(7) $$SL_{0}=R\times K\times L\times S$$

(8) $$SL_{t}=R\times K\times L\times S\times C\times P$$

(9) $$SD=SL_{0}-SL_{t}$$

Where *SL_0_* is the potential soil erosion intensity (t/(km²·a)), *SL_t_* refers to the actual soil erosion intensity (t/(km²·a)), and *SD *(t/(km²·a)) is the soil conservation amount.

### 2.5 Geodetector

Soil erosion intensity and soil conservation function were influenced by various factors and their interactions, and the Geodetector was used to detect their contribution to soil erosion intensity and soil conservation function. The *q* value can be obtained based on the following Equation 10 (Wang and Xu, 2017):


(10) $$q=1-\frac{\sum_{h=1}^{L}N_{h}\sigma_{h}^{2}}{N\sigma^{2}}$$

Where *q* is the explanatory ability of the independent variable X regarding the spatiotemporal variation of the dependent variable Y, with a range of 0-1. The closer of Y is to 1, the more significant its spatial variability. Moreover, *L* refers to the variance of Y values in stratum *h* and the whole region, respectively. Furthermore, interaction detection analyzes the combined effects of factors on Y by examining their interactions.

In addition, interaction detection analyzes the *q* values (*q* (X1) and *q* (X2)) to the interaction *q* value (*q* (X1∩X2)) effects to identify their interaction. The types of interactions include nonlinear weakness (*q* (X1∩X2)) < Min(*q* (X1), *q* (X2)), single-factor nonlinear weakness (Min(*q* (X1), *q* (X2)) < *q* (X1∩X2) < Max(*q* (X1), *q* (X2))), two-factor enhancement (*q* (X1∩X2) > Max(*q* (X1), *q* (X2))), independent (*q* (X1∩X2)= *q* (X1)+ *q* (X2)), and nonlinear enhancement (*q* (X1∩X2) > *q* (X1) + *q* (X2)).

## 3 Results

### 3.1 Spatiotemporal changes in actual soil erosion intensity

Based on the “Soil Erosion Intensity Classification Standards” (SL190-2008), the calculated actual soil erosion intensity were classified into six levels, including slight erosion (0–200 t/(km²·a)), mild erosion (200–2500 t/(km²·a)), moderate erosion (2500–5000 t/(km²·a)), severe erosion (5000–8000 t/(km²·a)), very severe erosion (8000–15000 t/(km²·a)), and extreme erosion (>15000 t/(km²·a)).

**Figure 3:**
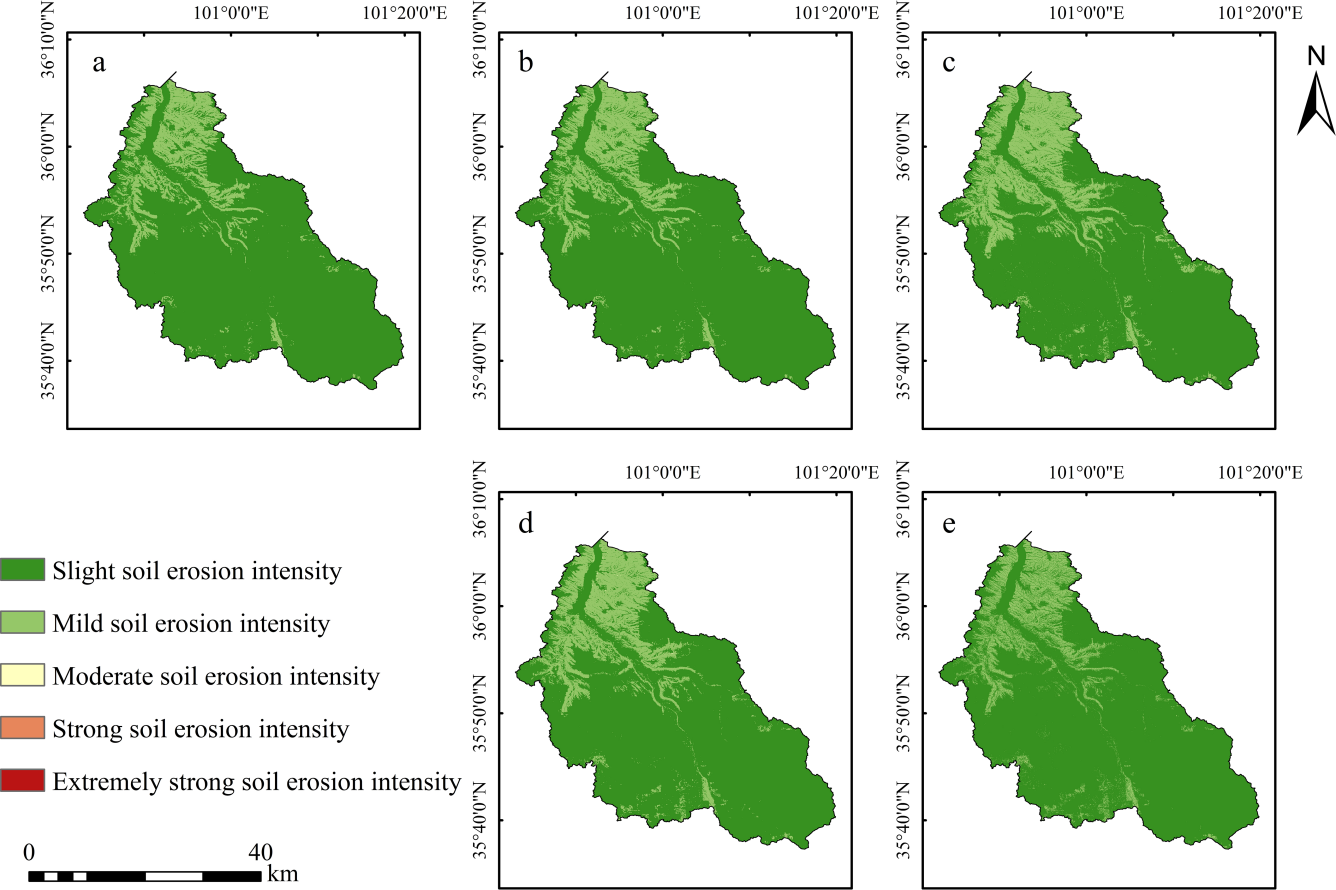
Spatial distributions of actual soil erosion intensity in the Shagou River watershed from 1980 to 2020. (**a**:1980, **b**: 1990, **c**: 2000, **d**: 2010, **e**: 2020).

During the study period from 1980 to 2020, the actual soil erosion intensity in the Shagou River watershed was predominantly characterized by slight erosion intensity (**Figure 3**). In 1980, slight erosion was mainly concentrated in the middle and lower reaches of the watershed, often distributed in a strip-like pattern along the river channels (**Figure 3a**). Moderate erosion was observed at the river confluence areas, while slight erosion near the river inlet displayed a distinct block-like aggregation. The upper reaches of the watershed were predominantly affected by mild erosion, with moderate erosion following a strip-like pattern along the river channels, and sporadic slight erosion distributed at the watershed’s boundaries and at the uppermost parts of the main river channels.

In 1990, the extent of actual soil erosion intensity showed little change (**Figure 3b**). The slight erosion in the upper reaches, along the main river channels and the watershed boundary, degraded into mild erosion over a very small area, while the extent of slight erosion in the middle and lower reaches expanded.

By 2000 (**Figure 3c**), compared to 1990, the range of slight erosion intensity along the main river channels in the upper, middle, and lower reaches, as well as along the eastern bank near the river inlet, had significantly expanded, with a predominant block-like aggregation. Some areas also exhibited a trend of transitioning from strip-like aggregation to block-like aggregation.

In 2010 and 2020 (**Figures 3d, e**), the actual soil erosion intensity extent showed marked improvement. In 2010, the range of slight erosion intensity decreased substantially, with block-like slight erosion intensity in the middle and lower reaches along the river channels degrading into sparse strip-like patterns. Most of the slight erosion intensity in the upper reaches, along both the eastern and western banks, also degraded into mild erosion intensity. By 2020, all the strip-like aggregated slight erosion intensity in the middle and lower reaches along the river channels had transformed into mild erosion intensity, further reducing the extent of slight erosion intensity. In the upper reaches, only scattered slight erosion intensity remained along the watershed boundary and the main river channels.

**Figure 4:**
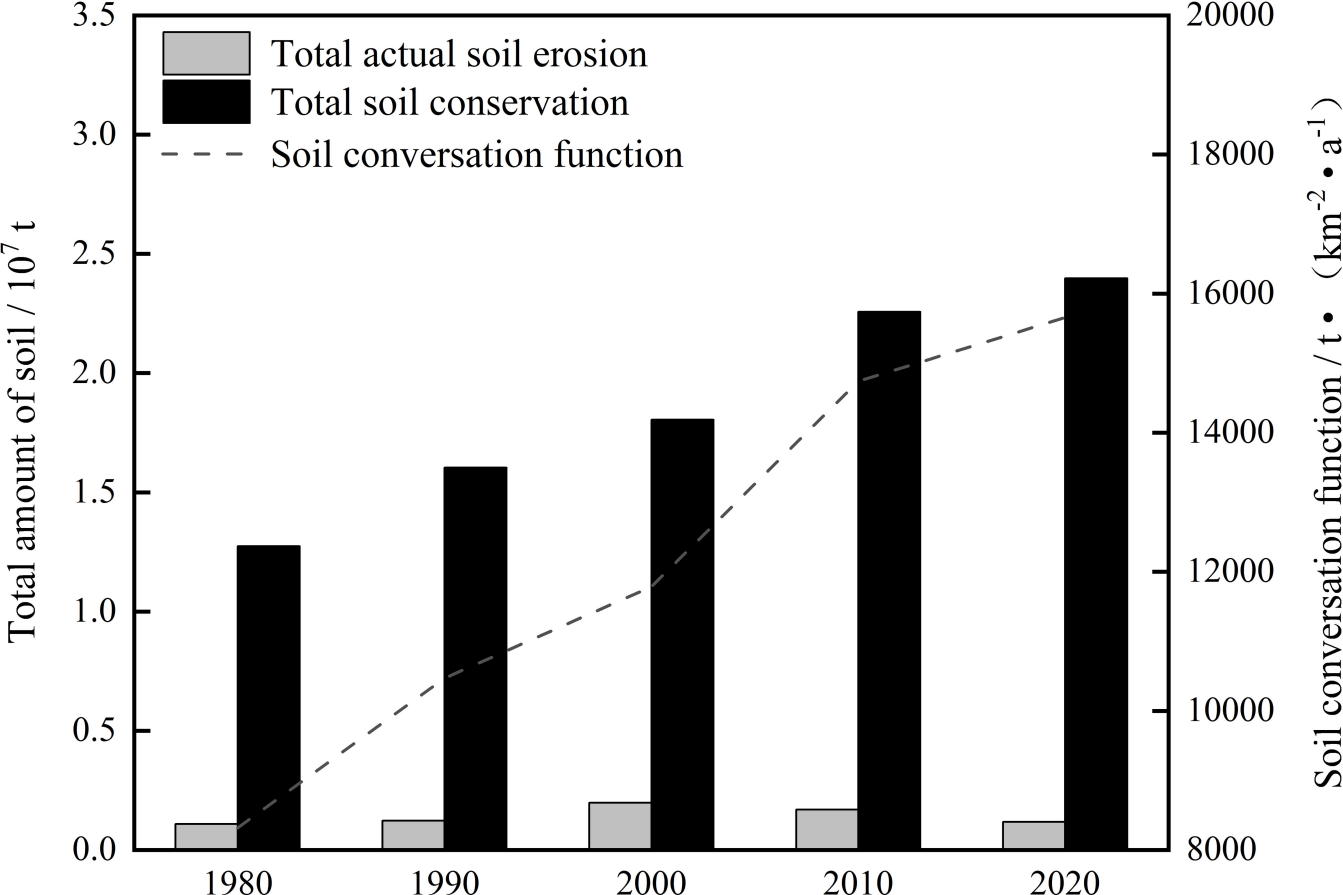
Total actual soil erosion and soil conservation function in the Shagou River watershed from 1980 to 2020.

Temporally, the actual soil erosion intensity status in the Shagou River watershed showed an evolution trend of first increasing and then decreasing (**Figure 4**). The year 2000 marked the peak of this evolution process, with the total actual soil erosion intensity reaching 0.20 × 10⁷ t and an erosion intensity of 1292.21 t/(km²·a). In contrast, in 1980, the actual soil erosion intensity level was at its lowest, with a total erosion amount of 0.11 × 10⁷ t and an intensity of 712.35 t/(km²·a). From 1980 to 2000, the total actual soil loss in the watershed increased from 0.11 × 10⁷ t to 0.20 × 10⁷ t, with a net increase of 887,939.47 t, corresponding to a growth rate of approximately 81.40%. From 2000 to 2020, however, the actual soil loss showed a significant decline, with a total reduction of 800,507.51 t, representing a decrease of 40.45%.

In conclusion, the year 2000 served as a critical turning point for the actual soil erosion intensity trend in the Shagou River watershed. Since then, the soil erosion status in the watershed has been continuously improving in a positive direction, reflecting a trend of ecological restoration and environmental optimization.

#### 3.1.1 Effect of altitude on actual soil erosion intensity

**Figure 5:**
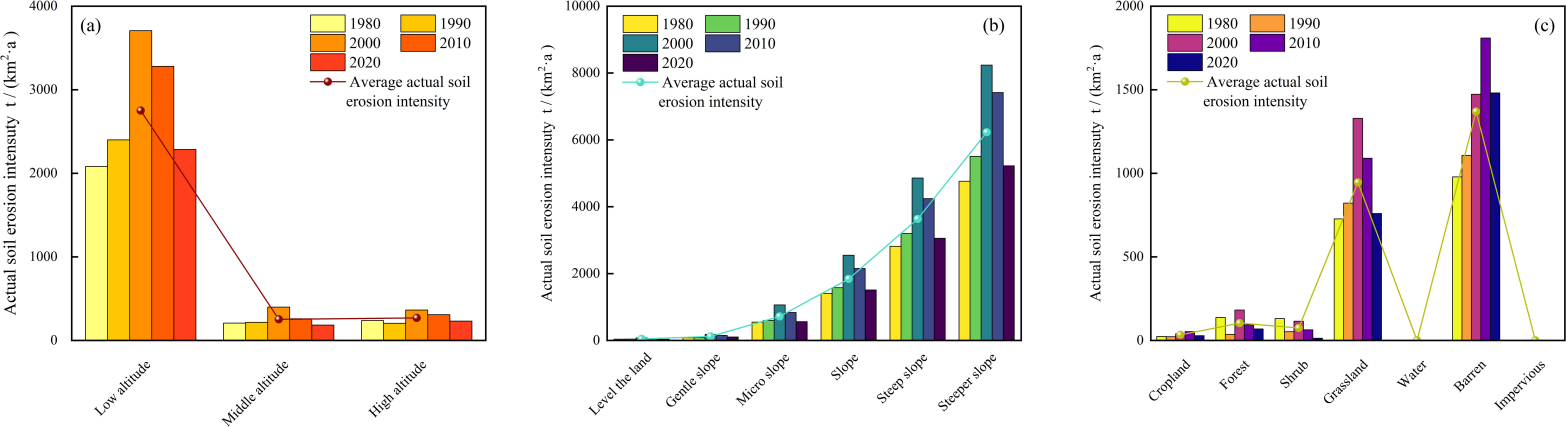
Actual soil erosion intensity at different elevations **(a)**, slopes **(b)**, and land use types **(c)** from 1980 to 2020.

Based on the actual elevation conditions in the watershed, geographic information system spatial analysis techniques were applied to reclassify the elevation data into three gradients: low altitude (<3000m), medium altitude (3000-3500m), and high altitude (>3500m) (**Figure 5a**).

The results indicate significant differences in actual soil erosion intensity across different elevation regions within this watershed. The greatest soil erosion intensity occurred in the low-altitude areas, followed by the high-altitude areas, with the weakest erosion intensity in the medium-altitude areas. Over the nearly 40-year study period, the actual soil erosion intensity from high to low was as follows: low-altitude areas (2750.85 t/(km²·a)), high-altitude areas (269.12 t/(km²·a)), and medium-altitude areas (252.80 t/(km²·a)).

During the period from 1980 to 2020, the actual soil erosion intensity in different elevation regions exhibited different temporal trends. In the low- and medium-altitude areas, soil erosion intensity initially increased and then decreased, while in the high-altitude areas, the soil erosion intensity exhibited a complex pattern of first decreasing, then increasing, and subsequently decreasing again. Notably, the change in erosion intensity in the medium-altitude areas was the most significant, with the actual soil erosion intensity in 2000 increasing by 92.34% compared to 1980, and decreasing by 53.83% compared to 2020.

#### 3.1.2 Effects of slope on actual soil erosion intensity

Based on the technical specifications in the “Technical Regulations for Slope Classification Map of Land Survey: TD/T1072-2022”, the slope of the watershed was classified into six categories, including flatland (<2°), gentle slope (2°-8°), slight slope (8°-15°), moderate slope (15°-25°), steep slope (25°-35°), and very steep slope (>35°).

The results indicate a significant positive correlation between actual soil erosion intensity and slope. As the slope gradually increases, soil erosion intensity increased, with the most notable changes observed in the steep and very steep slope regions (**Figure 5b**). Precise measurements revealed the following soil erosion intensities for each slope category: very steep slope (6228.73 t/(km²·a)), steep slope (3634.20 t/(km²·a)), moderate slope (1839.09 t/(km²·a)), slight slope (720.42 t/(km²·a)), gentle slope (119.06 t/(km²·a)), and flatland (46.49 t/(km²·a)).

Throughout the study period, actual soil erosion intensity in different slope categories exhibited an initial increase followed by a decrease. From 1980 to 2000, the most significant increase in soil erosion intensity was observed in the gentle slope region, which rose by 123.00% over the 20-year period. Significant increases were also observed in the slight slope, flatland, and moderate slope regions, with increases of 94.02%, 93.29%, and 81.12%, respectively. In contrast, the increases in the very steep slope (72.88%) and steep slope (72.74%) regions were relatively smaller. From 2000 to 2020, the decline in soil erosion across different slope types was as follows, from lowest to highest: very steep slope (36.56%) < steep slope (37.11%) < moderate slope (40.80%) < gentle slope (43.31%) < slight slope (47.03%) < flatland (53.12%).

#### 3.1.3 Effects of land use types on actual soil erosion intensity

As shown in **Figure 5c**, there were significant differences in the actual soil erosion intensity across different land use types. The erosion intensities followed the sequence, i.e., bare land suffered the biggest value of 1369.49 t/(km²·a), followed by grassland (945.40 t/(km²·a)), forestland (103.55 t/(km²·a)), shrubland (74.80 t/(km²·a)), and cropland (33.29 t/(km²·a)).

From 1980 to 2000, dynamic monitoring revealed that actual soil erosion intensity in cropland, forestland, and shrubland followed a similar trend, initially decreasing and then gradually increasing. In contrast, the soil erosion intensity in grassland and bare land showed a continuous intensification. Between 2000 and 2020, the soil erosion intensity in forestland, shrubland, and grassland exhibited a continuous decline, while the soil erosion intensity in cropland and bare land initially increased and then decreased.

Over the 20-year period, despite considerable differences in actual soil erosion intensity across land use types, the overall trend for all land use types showed a reduction in soil erosion. This finding is of significant scientific value for further understanding the dynamic relationship between land use and soil erosion intensity within this watershed.

### 3.2 Characteristics of spatiotemporal changes in soil conservation function

A steady increase in the soil conservation function occurred in the Shagou River watershed (**Figure 4**). The corresponding soil conservation functions increased from 8317.72 t/km²·a in 1980, 10470.86 t/(km²·a) in 1990, 11786.62 t/(km²·a) in 2000, 14739.62 t/(km²·a) in 2010, and to 15657.49 t/(km²·a) in 2020. In contrast to the actual ones, the total amount of soil conservation reached its maximum value of 2.40 × 10⁷ t in 2020, while the minimum value of 1.27 × 10⁷ t was recorded in 1980. A further analysis of the change rates of soil conservation across various periods showed that the most significant growth took place between 1980 and 1990, with a growth rate of 25.89%. In contrast, the period from 2010 to 2020 witnessed the lowest growth rate, which was merely 6.23%.

**Figure 6:**
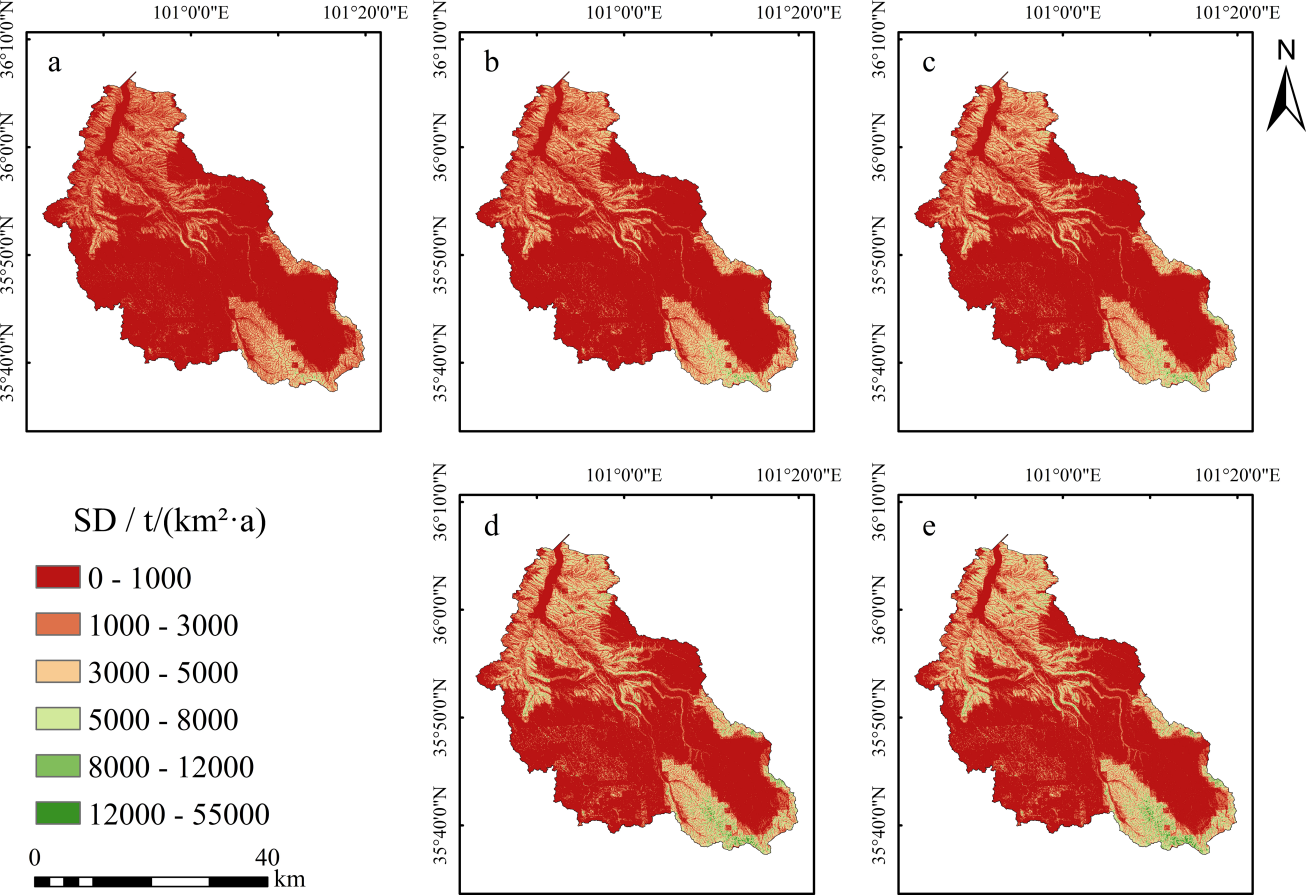
Maps of the spatial distributions of soil conservation function in the Shagou River watershed over the past 40 years (**a**:1980, **b**: 1990, **c**: 2000, **d**: 2010, **e**: 2020) and (SD: the soil conservation amount).

Spatially, the pattern of soil conservation function in the middle reaches of the watershed remained relatively stable over the 40 years (**Figure 6**). However, significant changes were observed in the upper reaches along the main river channels, this watershed boundaries, and the lower reaches along the river course. Specifically, the areas of high soil conservation function (>5000 t/(km²·a)) along the eastern bank near the river inlet and in the upper reaches expanded significantly. The originally sparse linear aggregation areas gradually evolved into continuous block-like regions. In summary, over the past 40 years, the total soil conservation in the Shagou River watershed increased by 1.12 × 10⁷ t, with a growth rate of 88.24%.

#### 3.2.1 Effects of altitudes on soil conservation function

**Figure 7:**
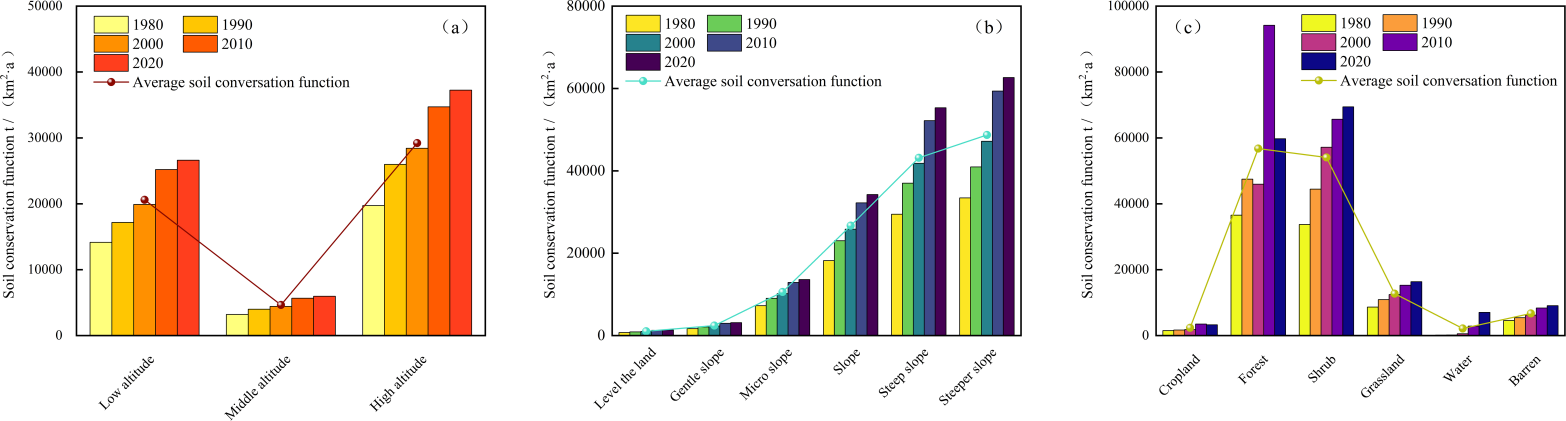
Soil conservation function on different elevations **(a)**, slopes **(b)**, and land use types **(c)** from 1980 to 2020.

In the study area, significant differences were observed across different periods and elevation conditions (**Figure 7a**). The soil conservation function was more prominent in high-altitude areas, and the soil conservation function was ranked from the highest to the lowest as follows: high altitude (29218.29 t/(km²·a)), low altitude (20606.33 t/(km²·a)), and medium altitude (4646.25 t/(km²·a)).

From the internally perspective, the soil conservation function in the low, medium, and high-altitude regions all exhibited an increasing trend. Notably, the growth trend in the high-altitude areas was particularly significant, with a growth rate of 88.81% compared to 1980. The growth rate in low-altitude areas was 88.23%, and in medium-altitude areas, it was 86.61%.

#### 3.2.2 Effects of slopes on soil conservation function

In the study area, there was a significant positive correlation between soil conservation function and slope (**Figure 7b**). Specifically, the growth rate of soil conservation function was exceptionally rapid in several slope intervals, including from gentle slope to slight slope, slight slope to moderate slope, and gentle slope to flatland, with growth rates exceeding 100%. The soil conservation function was the highest on very steep slopes [48712.19 t/(km²·a)], followed by steep slopes [43159.99 t/(km²·a)], moderate slopes [26700.38 t/(km²·a)], slight slopes [10587.03 t/(km²·a)], gentle slopes [2431.13 t/(km²·a)], and flatland exhibited the lowest value [1042.71 t/(km²·a)].

During the study period, the moderate slope region exhibited the most significant increase in soil conservation function, with a growth rate of 88.39% In other regions, the growth rates decreased from steep slope (87.81%), very steep slope, (87.35%), slight slope (87.15%), gentle slope (87.08%), and the flatland (85.97%).

#### 3.2.3 Effects of land use types on soil conservation function

There were significant differences in soil conservation function across various land types within the study area (**Figure 7c**). Forestland had the highest value [56776.72 t/(km²·a)], followed by shrubland [54089.59 t/(km²·a)], grassland [12716.32 t/(km²·a)], bare land [6721.37 t/(km²·a)], and cropland had the lowest value [2348.12 t/(km²·a)].

Between 1980 and 2020, the soil conservation function in all land types exhibited an upward trend. Notably, the changes in cropland and shrubland were the most significant. Over nearly 40 years, soil conservation function in cropland increased by 116.49%, and in shrubland, it increased by 105.94%. Following these, the increases in bare land, grassland, and forestland were also notable, with bare land increasing by 98.68%, grassland by 89.23%, and forestland by 63.43%. This upward trend highlights the evolving role of different land use types in soil conservation function and provides valuable insights for ecological restoration and soil conservation planning.

### 3.3 Analyses of driving factors

#### 3.3.1 Driving factors of actual soil erosion intensity

**Figure 8:**
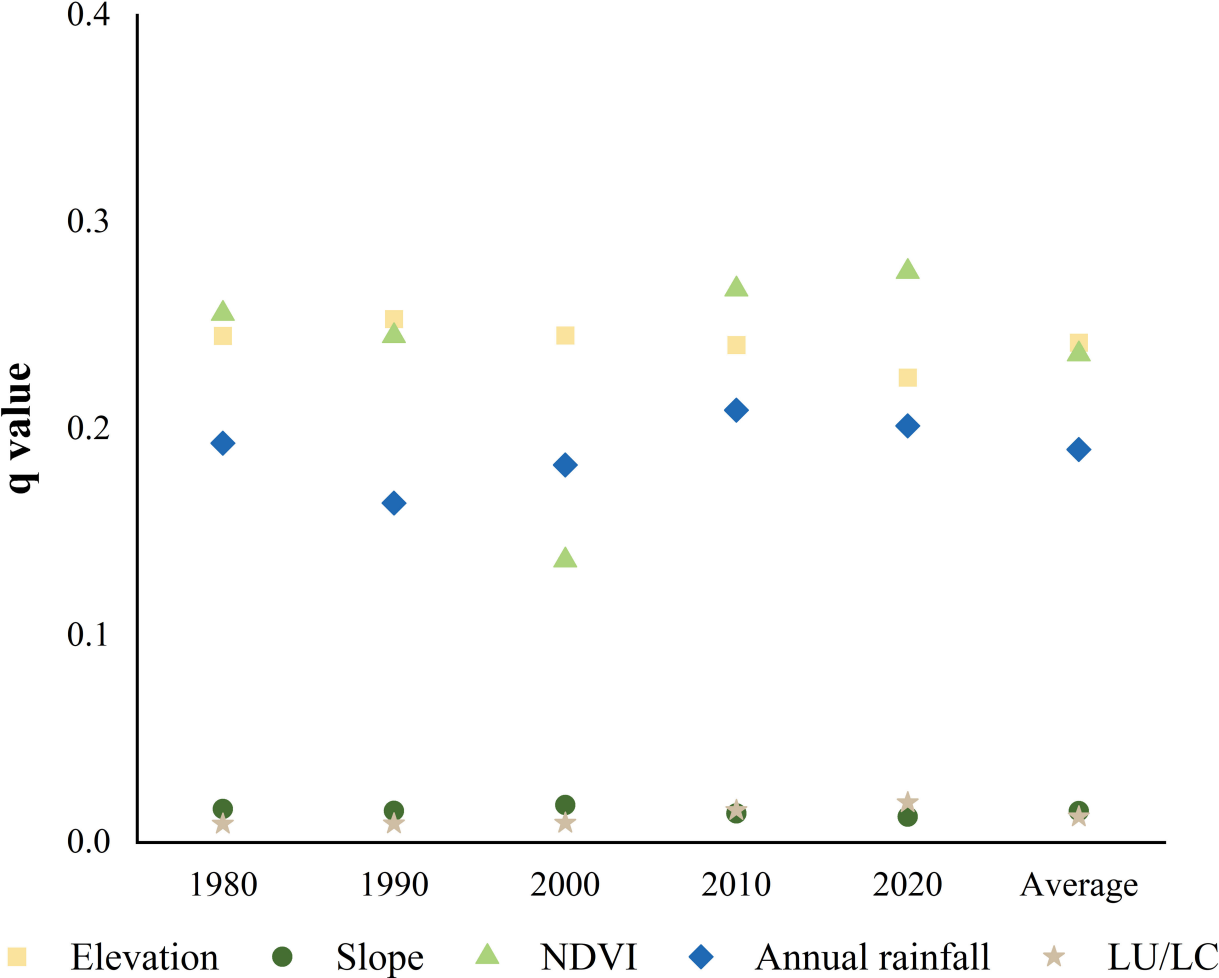
The *q*-values of factors influencing actual soil erosion intensity.

Based on the average *q*-values across the five time periods (**Figure 8**), the elevation ranked the first (*q* = 0.24), followed by vegetation coverage (*q* = 0.24) and annual rainfall (*q* = 0.19). Slope (*q* = 0.02) and land use type (*q* = 0.01) had relatively weaker effects. Notably, all the five factors passed the significance test, indicating their indispensable roles in the soil erosion process.

From 1980 to 2020, the elevation consistently served as the dominant factor explaining the spatial distribution of actual soil erosion intensity, while land use type exhibited relatively limited explanatory power. A detailed analysis revealed that the influence of elevation and slope gradually diminished over time. In contrast, the explanatory power of vegetation coverage, annual rainfall, and land use type steadily increased, highlighting their growing importance in shaping the patterns of soil erosion.

**Figure 9:**
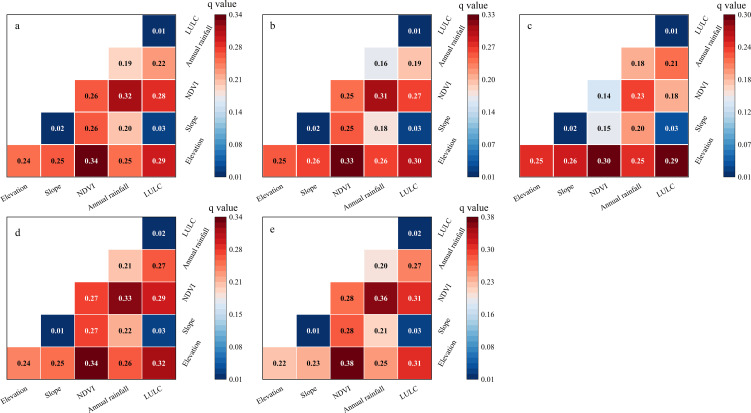
Interaction detection results of actual soil erosion intensity influencing factors in the Shagou River watershed from 1980 to 2020. (**a**:1980, **b**:1990, **c**:2000, **d**:2010, and **e**:2020).

Further examination of interactions among the factors revealed significant bivariate enhancement effects across the combined factors (**Figure 9**). The synergistic effect of “elevation ∩ vegetation coverage” was the most influential, with its *q*-value peaking at 0.38 in 2020. This made it the key driver of the spatial distribution of soil erosion intensity, followed by the combinations of “vegetation coverage ∩ annual rainfall” and “elevation ∩ land use type”, which collectively regulated the occurrence and development of soil erosion intensity to varying degrees. At the watershed scale, interactions dominated by elevation and vegetation coverage exhibited the strongest explanatory power when combined with other factors (**Figure 9**).

#### 3.3.2 Driving factors of soil conservation function

**Figure 10:**
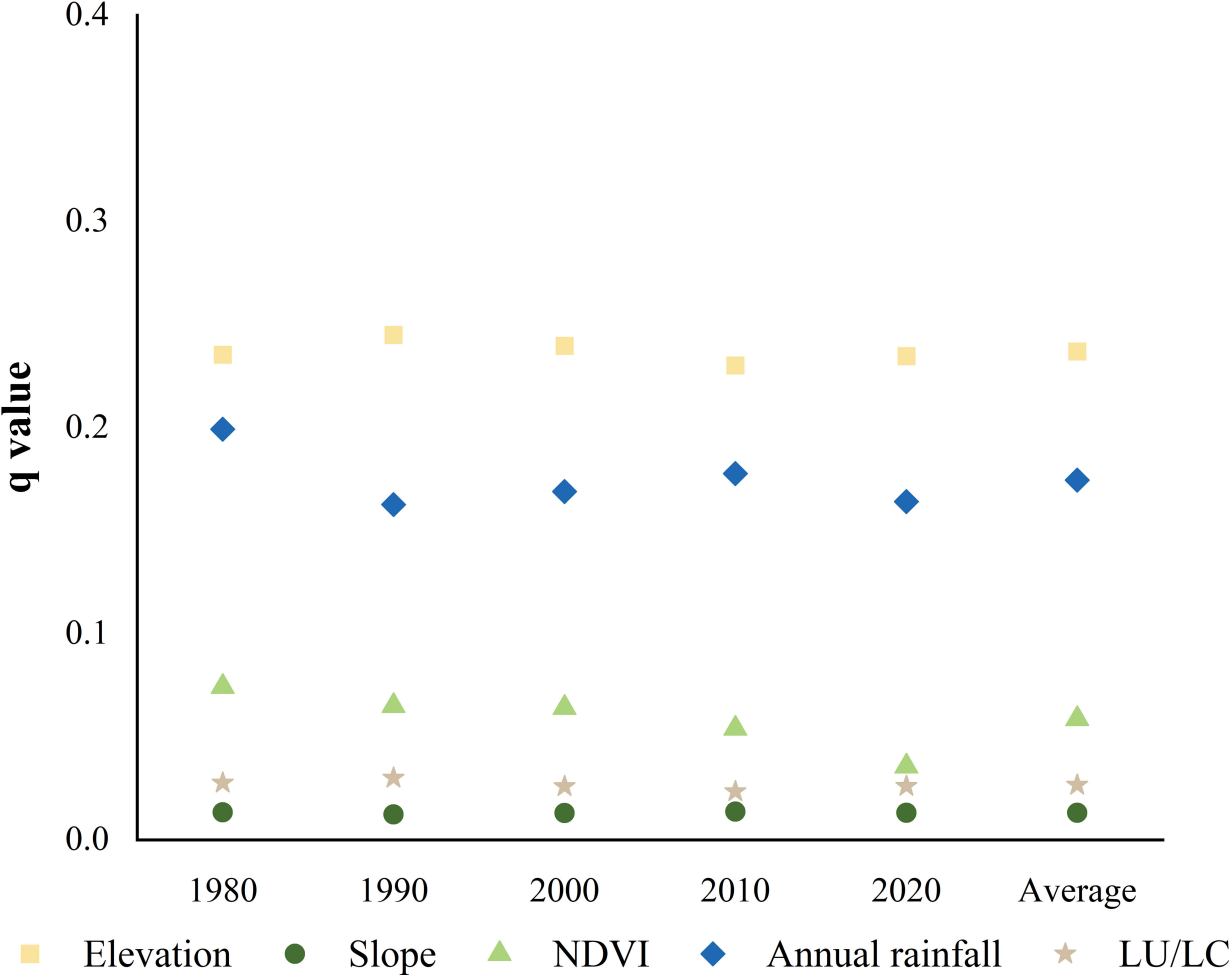
The *q*-values of factors influencing soil conservation function.

In this study, the driving factors of soil conservation function were thoroughly analyzed using the Geodetector. **Figure 10** demonstrated that the average *q*-values of the factors were the lowest for the slope (0.013), followed by land use type (0.027), vegetation coverage (0.059), and annual rainfall (0.175). The elevation played a more prominent role in soil conservation function with the highest value of 0.237.

**Figure 11:**
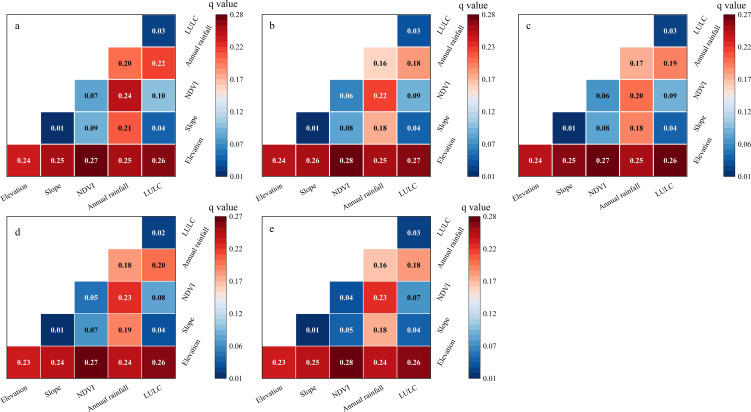
Interactive detection results of soil conservation function influencing factors from 1980 to 2020. (**a**: 1980, **b**:1990, **c**:2000, **d**:2010, and **e**:2020).

All the five factors exhibited significant bivariate enhancement relationships (**Figure 11**). Specifically, the combined effect of these factors on soil conservation function was not a simple linear sum but generated synergistic enhancement effects. Among the combinations, the combinations of the elevation factor with other factors exhibited the most significant explanatory power. This provides crucial insights and theoretical support for accurately interpreting the complex causes and dynamic changes in soil conservation function. The findings offer significant guidance and practical value for maintaining soil ecosystems and for scientifically soil protection and management strategies.

## 4 Discussion

Soil erosion intensity and soil conservation function serve as two crucial indicators for assessing ecosystems and guiding the prevention and control of land degradation. In this study, InVEST model was employed to evaluate the soil erosion intensity and soil conservation function in the Shagou River watershed from 1980 to 2020. The average soil erosion intensity was found to be 936 t/(km²·a), while the average soil conservation function reached 12194 t/(km²·a). These results are in line with the findings of previous studies (Chen et al., 2020; Kang et al., 2018), which effectively validates the reliability of the outcomes of this study.

From 1980 to 2020, the soil erosion intensity steadily increased that is consistent with previous conclusions (Cen et al., 2023; Hou et al., 2024; Li et al., 2023; Yan et al., 2023; Yang et al., 2024). However, certain areas still exhibit weak soil conservation function. This suggests that the potential risk of soil erosion in this watershed is still high, likely due to intensified soil erosion resulting from high frequency of precipitation and low vegetation cover.

In this study, higher soil conservation function occurred in woodland, shrubland, and grassland types that was consistent with assertions by previous studies (Kumar et al., 2023). This may be attributed to high vegetation cover in the low-elevation areas. Additionally, the root systems of forest land are more developed compared to those of grasslands, resulting a greater soil conservation function in the forest land. Conversely, the highest intensity of soil erosion in bare land areas with loose sediment texture impedes moisture conservation and renders high erosion (Shen et al., 2024).

Soil erosion intensity is influenced by both natural factors and human activities. The over-exploited natural resources and unregulated urbanization, as well as rising temperatures and precipitation had contributed to a significant increase in soil erosion intensity within the Shagou River watershed. Fortunately, emphasis on ecological protection and public awareness of environmental conservation has increased in recent years. A series of ecological protection projects have been carried out to reduce soil erosion. These include converting farmland into forests and grasslands, as well as sand prevention and control initiatives. The significance of sustainable development has also been emphasized. Consequently, the total amount of soil erosion in the study had reduced by 40.45% from 2000 to 2020, resulting in improved soil conservation function across the study period.

**Figure 12:**
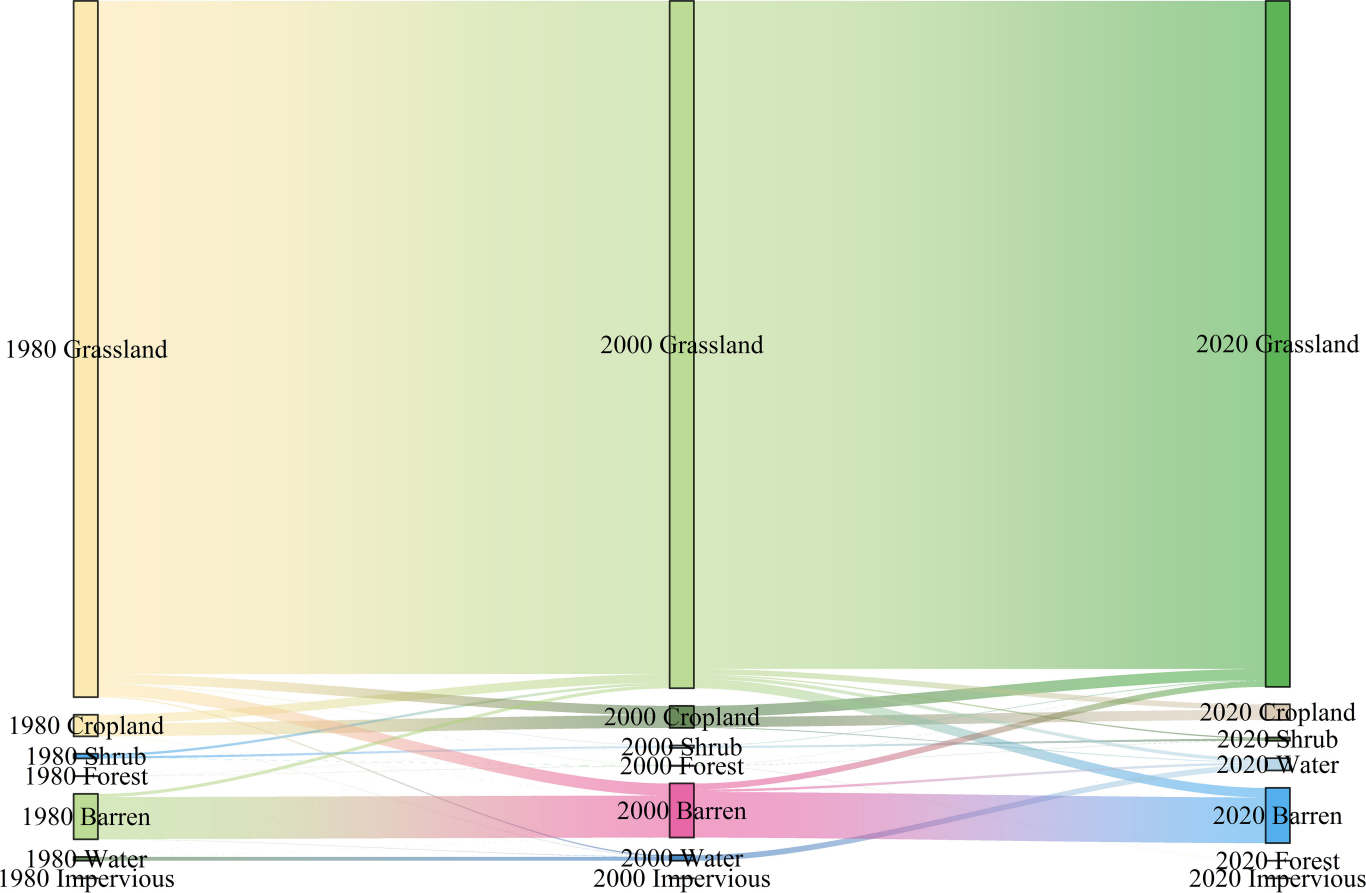
Characteristics of land use type shifts in the Shagou River watershed from1980 to 2020.

The upper reaches of the Yellow River Basin are situated on the ecologically fragile Tibetan Plateau, where they confront substantial environmental challenges. Accelerated global warming, rising atmospheric humidity, and expansive human activities have collectively driven elevated surface runoff volumes. This hydrological transformation has triggered a worrying shift in land cover dynamics, where vegetated zones are progressively being converted into human settlements and barren landscapes (**Figure 12**). Such land-use changes have intensified soil erosion processes, significantly diminishing the region’s natural soil retention capacity. To address these soil degradation issues in the Shagou River watershed and comparable areas, stakeholders should prioritize enhanced monitoring systems for land cover transformations and land-use typologies. Targeted interventions should include the establishment of designated soil erosion control zones in vulnerable areas. Concurrently, regulatory frameworks must enforce strict controls over new land-use applications, including rigorous evaluations of project types and scale. Equally critical is the enforcement of prohibitions against environmentally disruptive activities that exacerbate erosion risks. These combined measures aim to balance developmental needs with ecological preservation through proactive land management and sustainable resource utilization strategies.

It should be noted that this study may involve certain limitations. Specifically, the simulation employed default parameters for the study period and maintained constant *K* values throughout. Furthermore, the *P* values were adopted from prior research, which could potentially lead to computational inaccuracies. Nevertheless, it is crucial to underscore that the yield outcomes derived from this study remain plausible and offer meaningful contributions. These findings can serve as a valuable reference for both academic exploration and practical implementations within the upper alpine region of the Yellow River basin.

## 5 Conclusions

In this study, the InVEST model was employed to analyze the soil erosion and soil conservation function, and the driving factors were identified using Geodetector for the typical alpine watershed in the upper Yellow River during 1980-2020. The results showed that soil erosion intensity in the Shagou River watershed steadily increased from 721.48 t/(km²·a) to a 2000 peak of 1292.49 t/(km²·a), an 81.40% rise from 1980 to 2000. After 2000, it declined, reaching 769.63 t/(km²·a) in 2020, a 40.45% drop, due to ecological restoration. Furthermore, the most severe erosion occurred in low - altitude areas (below 3000 m) and on steep slopes (greater than 35°). Bare land exhibited the highest soil erosion intensity, measuring 1369.49 t/(km²·a), whereas cultivated land had the lowest, at just 33.29 t/(km²·a).

Between 1980 - 1990, soil conservation function grew most, at 25.89%, while 2010–2020 had the lowest rate, 6.23%. Over the past 40 years, the Shagou River watershed’s soil conservation function improved substantially, with a 1.12×10⁷ t increase. The upper and middle reaches, especially upstream mountains, saw the biggest rises. Soil conservation function varied by period, altitude, and slope. High - altitude [29,218.29 t/(km²·a)] and steep [48,712.19 t/(km²·a)] areas were most effective, while middle - altitude [4646.25 t/(km²·a)] and flat [1042.71 t/(km²·a)] areas were less so. Forest land was key for soil conservation, and cultivated land had the weakest capacity.

In this watershed, elevation and vegetation cover were the main drivers of soil erosion intensity, with land use type having less impact. The influence of elevation declined, while that of vegetation cover and annual precipitation grew. Elevation and vegetation cover, especially in interaction with other factors, were dominant, with elevation being the core factor for soil conservation function. Slope had the weakest explanatory power for soil conservation function, and the interaction of elevation with other factors had the most significant effects.

This study shows that ecological restoration and soil - water conservation measures in the watershed have had long - term positive impacts, particularly in the upper - reach key ecological protection areas. Through enhanced vegetation restoration and slope - related conservation efforts, soil erosion intensity has been notably reduced, and soil conservation function has improved. These measures not only boost total soil conservation function in the watershed but also foster the restoration and stability of its ecosystem.

## Data Availability

The original contributions presented in the study are included in the article/supplementary material. Further inquiries can be directed to the corresponding author.
